# Transcriptome meta-analysis of Kawasaki disease in humans and mice

**DOI:** 10.3389/fped.2024.1423958

**Published:** 2024-09-16

**Authors:** Wanjun Gu, Sarah Mirsaidi-Madjdabadi, Francisco Ramirez, Tatum S. Simonson, Ayako Makino

**Affiliations:** ^1^ Department of Medicine, University of California, San Diego, CA, United States; ^2^ Center for Inflammation Science and Systems Medicine, The Herbert Wertheim University of Florida Scripps Institute for Biomedical Innovation & Technology, Jupiter, FL, United States

**Keywords:** Kawasaki disease, transcriptomics, RNA sequencing, meta-analysis, animal model

## Abstract

Kawasaki Disease (KD) affects young children less than five years old with severe blood vessel inflammation. Despite being treatable, the causes and mechanisms remain elusive. This study conducted a meta-analysis of RNA sequencing (RNA-seq) data from human and animal models to explore KD's transcriptomic profile and evaluate animal models. We retrieved bulk and single-cell RNA-seq data from Gene Expression Omnibus, with blood and coronary artery samples from KD patients, aorta samples from KD mouse models (*Lactobacillus casei* cell wall extract-injected mice), and their controls. Upon consistent quality control, we applied Fisher's exact test to assess differential gene expression, followed by an enrichment analysis of overlapping genes. These studies identified 400 differentially expressed genes in blood samples of KD patients compared to controls and 413 genes in coronary artery samples. The data from KD blood and KD coronary artery samples shared only 16 differentially expressed genes. Eighty-one genes overlapped between KD human coronary arteries and KD mouse aortas, and 67 of these 81 genes were regulated in parallel in both humans and mice: 30 genes were up-regulated, and 37 were down-regulated. These included previously identified KD-upregulated genes: *CD74*, S*FRP4*, *ITGA4*, and *IKZF1*. Gene enrichment analysis revealed significant alterations in the cardiomyopathy pathway. Single-cell RNAseq showed a few significant markers, with known KD markers like *S100A9*, *S100A8*, *CD74*, *CD14*, *IFITM2*, and *IFITM3*, being overexpressed in KD cohorts. Gene profiles obtained from KD human coronary artery are more compatible with data from aorta samples of KD mice than blood samples of KD humans, validating KD animal models for identifying therapeutic targets. Although blood samples can be utilized to discover novel biomarkers, more comprehensive single-cell sequencing is required to detail gene expression in different blood cell populations. This study identifies critical genes from human and mouse tissues to help develop new treatment strategies for KD.

## Introduction

Kawasaki Disease (KD) is a systemic vasculitis primarily affecting young children under the age of five years. Dr. Tomisaku Kawasaki first observed a patient with unknown unique symptoms with 50 more patients’ data in 1967, called an acute febrile mucocutaneous lymph node syndrome (1). This symptom was later named KD. Initially, the relation between KD and cardiac events was unclear. In 1970, the nationwide survey of KD in Japan documented several cases of cardiac arrest following KD onset (2). Today, coronary artery vasculitis and the eventual manifestations of coronary artery aneurysms (CAAs) are integral to the understanding of KD development in young children (3), and cardiac events are the primary cause of death in KD patients. In addition to CAAs, this disorder is characterized by a constellation of clinical symptoms, including prolonged high fever, bilateral conjunctival injection, oral mucosal changes, extremity changes, and cervical lymphadenopathy (4–6), many of which suggest an infectious etiology. Seasonality is one of the significant drivers of KD incidence, case clustering, and the occurrence of epidemics (7). However, the data also suggest that genetic predisposition plays a key factor (8–11). For example, significantly higher KD incidence rates are reported in East Asian countries, specifically Japan, relative to Western countries. The incidence of KD in East Asian countries is 10–30 times higher than the incidence in the United States and Europe, and it affects males at 1.5 times the rate of females globally (12). In addition, in Western countries like the United States, KD is much more prevalent in populations of Japanese descent. Familial accumulation of the disease in siblings supports the hypothesis that KD development has genetic components (13).

Other studies suggest the biological mechanisms underlying the pathogenesis of this disease are directly related to immune function. B lymphoid tyrosine kinase (BLK), a protein involved in B-cell activation, is significantly increased in mononuclear cells of acute KD patients (14). The T allele of SNP rs2736340 has been shown to lower the expression of BLK in blood B cells during the acute stage of KD, thus disrupting B cell function and increasing susceptivity to KD (15). The *CASP3* gene encodes for the caspase-3 protein, which regulates immune response and T-cell apoptosis. The rs72689236 SNP variant is associated with an increased risk of KD because it prevents binding with NFAT (Nuclear Factor of Activated T-Cells), a transcription factor that regulates *CASP3* expression in immune cells. Changes in *CASP3* expression (8, 10) could increase one's susceptibility to KD due to alterations in immune response. CD40 is a cell surface receptor that interacts with the CD40 ligand to activate B and T cells. The rs4813003 SNP in CD40 increases the risk of KD in Chinese populations by activating immune cells (16). *FCGR2A* encodes an immunoglobin Fc region. The AA genotype of the rs1801274 SNP of *FCGR2A* correlates with KD occurrence due to enhanced binding affinity of immune cells to IgG antibodies, leading to excessive inflammation (17, 18). The C allele of *ITPKC* has been shown to disrupt calcium signaling in T-cells, thus contributing to the immune hyper-reactivity of KD and correlating to higher KD susceptibility (19). Genetic variation in the gene *SMAD3*, which aids in downregulating T-cells via TGF-β signaling pathways, has been shown to increase KD susceptibility with developing inflammation and CAAs (20). Other genes, such as *TNF*, *IL-1*, *IL4*, and *HLA* (21–23), are also speculated to be involved in KD development, but more research is needed to confirm and understand the specific SNPs of these genes linked to KD.

Integrative transcriptomic analyses have elucidated the role of non-coding RNAs, such as microRNAs and long non-coding RNAs, as potential biomarkers for KD (21). Proteomics and metabolomics studies also identified potential KD biomarkers (22, 23). Leucine-rich alpha-2-glycoprotein (LRG1) was determined to be a reliable protein biomarker whose levels were elevated in acute KD patients (23), and the tryptophan metabolic pathway was significantly altered in KD patients and thus could serve as a diagnostic biomarker (22).

Recent research highlighted the potential contribution of pre- and post-natal risks to KD. Environmental factors like increased exposure to high levels of air pollution (carbon monoxide, nitric oxide, nitric dioxide, and nitrogen oxide) during, pre-, or post-birth in young children were found to be positively associated with KD development, according to Taiwan's National Health Insurance Research Database (24). Based on several studies conducted in Japan and Washington State, maternal influences such as smoking and geriatric pregnancy (over the age of 35) increased the risk of KD development in young children (25, 26). Furthermore, children exposed to smoking between 6 and 18 months of age were at higher risk of developing KD (26). The Washington study and Japan-based survey have found that infants hospitalized for bacterial infections or neonatal sepsis are more likely to develop KD later in childhood (25, 27). A Japanese nationwide survey showed children who were born pre-term (22–36 weeks) are shown to be at a higher risk for developing KD in comparison to those who went to term (37–41 weeks) (28).

Although extensive investigations are ongoing to identify the mechanisms of KD, the treatments for KD patients are limited. Intravenous immunoglobulin (IVIG) has been a common treatment to reduce the development of coronary arterial abnormality in children with KD within ten days of onset (29–31) alongside high-dose aspirin (80–100 mg/kg/day) (30, 31). Tumor necrosis factor-alpha blockers like infliximab are an alternative treatment for children who develop IVIG-resistant KD (32). Currently, researchers are exploring the potential of new therapies for KD, including interleukin-1 blockade treatment (33), statins (34), and plasma exchange (34), with promising results. However, the death after cardiac events is still high among KD patients. Therefore, there is a need to understand the mechanisms of KD further to develop novel and more efficient treatments for KD patients. Integrative studies systematically comparing animal models to human KD patients in terms of their phenotype and transcriptomic profile are also lacking. This study is thus designed to compare transcriptomic data within humans and between humans with KD and animal models of KD.

## Material and methods

### Data acquisition and exclusion

The deposited transcriptomic sequencing data on human Kawasaki Disease (KD) patients and *Lactobacillus casei* cell wall extract induced (LCWE) KD model mice were retrieved from Gene Expression Omnibus (GEO) (35). Although we have conducted an initial explorative analysis using all available data, some data could not be used in this analysis because the sample size/power was too low, and some mouse data were not well harmonized with human genes. For human data, RNA sequencing datasets with fewer than 20 samples were excluded to avoid potential biases and ensure adequate statistical power, aligning with established guidelines in transcriptomics research. Mouse datasets were excluded if they could not be adequately harmonized with human data, particularly if fewer than 50% of mouse genes could be reliably mapped to human orthologs using Ensembl. Additionally, mouse datasets were screened for high variability and non-overlapping expression profiles, as determined by principal component analysis. Mice datasets with high variability were excluded from the final analysis. Ultimately, we used the data from human RNA sourced from patients’ whole/peripheral blood and coronary arteries and mouse RNA collected from the abdominal aorta (Table 1).

**Table 1:** Gene expression omnibus database studies examined.

Study accession	Species	Samples	Assay method	Citation	In figures
GSE178491	Human	Whole blood	RNAseq	Ghosh et al. (36)	Used
GSE64486	Human	Coronary arteries	RNAseq	Rowley et al. (37)	Used
GSE200743	Human	Peripheral blood	scRNAseq	Chen et al. (38)	No
GSE152450	Human	Peripheral blood monocytes	scRNAseq	Geng et al. (39)	No
GSE168732	Human	Peripheral blood mononuclear cells	scRNAseq	Wang et al. (40)	Used
GSE141072	Mouse	Abdominal aorta	RNAseq	Porritt et al. (41)	Used
GSE178799	Mouse	Heart	scRNAseq	Porritt et al. (42)	No
GSE178765	Mouse	Abdominal aorta	scRNAseq	Porritt et al. (42)	Used

### Quality control of bulk and single-cell RNA sequencing data

Quality control procedures were homogenously conducted on all study datasets to ensure the consistency of bulk and single-cell RNA sequencing data. Genes with insufficient read counts (counts per million reads mapped, or CPM<100) were filtered out for bulk RNA sequencing data. All gene count matrices in the bulk RNA sequencing datasets were corrected for heteroskedasticity in gene expression using *limma* v3.1 (43). To minimize the influence of noise or background signal in single-cell RNA sequencing datasets, genes that appeared in no more than 0.1% of all the cells sequenced within each study were excluded, and cells must have expressed at least 200 different genes to be retained. The percentage of mitochondrial genes for each cell in the datasets was calculated and used as a metric of cell viability. To further remove low-quality cells or potential doublets from the analysis, cells with a unique gene count less than 250 or greater than 2,000 and cells with a mitochondrial gene percentage greater than 10% were also excluded from all single-cell RNA sequencing studies (44). All single-cell datasets were harmonized using *Seurat* v5.0 (45, 46).

### Differential gene expression analysis

For the bulk RNA sequencing datasets, Fisher's exact test was used to test for differential gene expression between the KD patients or KD mouse model and respective controls. Nominal *P*-values were then corrected using the Benjamini-Hochberg procedure to establish statistical significance based on false discovery rates (FDR). This approach was chosen to minimize the risk of Type I errors while accounting for the large number of genes tested. In both humans and mice, a gene is only considered to be significantly differentially expressed when the absolute value of its expression log fold change (LogFC) is greater than or equal to 1 (LogFC≥-1 or LogFC≥1), and the associated *P*-value, upon FDR correction, is smaller than or equal to 0.05. A gene is classified as commonly differentially expressed in both humans and mice if its expression meets the criteria of significance in both species (|LogFC|≥1 and FDR≤0.05). If a gene is significant only in humans and not in mice, it is designated as human-specific, and conversely, if significant only in mice, it is considered mouse-specific. For the single-cell RNA sequencing datasets, all cells that passed the quality control filters were divided into KD and non-KD cells, homogenized in-silico, and tested for differential gene expression. For both the bulk and single-cell analyses, the significant genes identified in mice were converted to their human homologs using the Ensembl database (47), and they were compared with differentially expressed genes in human studies to determine the overlap of these gene sets. Concordant set analysis was conducted to identify similar and distinctive gene expression signatures in human KD patients and KD animal models.

### Biological pathway analysis

Biological pathway analysis was conducted to identify pathways relevant to KD using a combination of established databases and tools. Pathways were selected based on their enrichment in differentially expressed genes (DEGs) identified in both human and mouse datasets as well as human or mouse datasets uniquely. Enrichment analysis was performed using the PathfindR package (48), applying a hypergeometric test to identify significantly enriched pathways (*P* < 0.05, adjusted for false discovery rate). Pathways with low relevance or lacking sufficient gene coverage were excluded to enhance the specificity of the analysis. Top Gene Ontology (GO) terms with the most significant *P*-value for enrichment and with the most genes involved were visualized.

## Results

Eight transcriptomic sequencing datasets deposited into GEO under the keyword “Kawasaki Disease” were included in the study. Initially, five studies were conducted in humans (36–40), and three studies were carried out in mice exposed to LCWE (33, 41, 42). We were able to use three of the five human data sets and two of the three mouse data sets (Table 1). The reason for the exclusion of some data was due to the low power to analyze the data statistically.

### Transcriptomic profile differences in coronary artery and whole blood in humans

**Figure 1:**
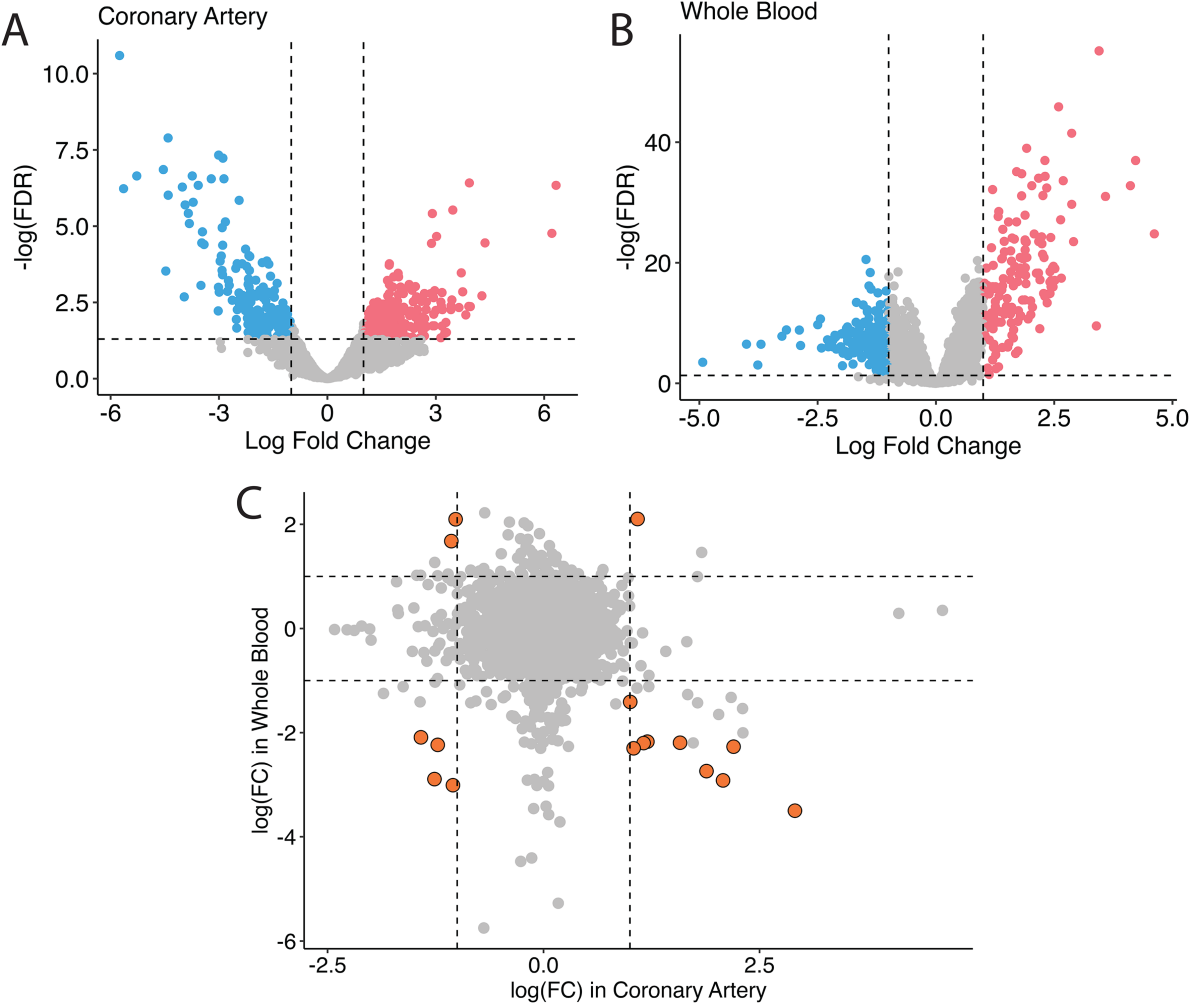
Bulk differential gene expression profile in humans. In humans, when overlapping the transcriptomic profile in coronary tissues **(A)** and in the blood **(B)**, fewer significant genes are found, and they non-specifically distribute across all four quadrants **(C)**.

In humans, studies of GSE178491 (whole blood) and GSE64486 (coronary arteries) were used to compare the gene expression profiles between different tissue types. In human whole blood, 2,500 genes were differentially expressed in KD vs. non-KD after correcting for false positive rate (Figure 1A), whereas in human coronary arteries, 428 genes were significantly differentially regulated (Figure 1B). Although many genes were differentially expressed in both tissue types, the transcriptomic profiles of KD patients in these different tissue types are homogeneous (Figure 1C). Among the 16 genes that are differentially expressed in both tissue types, only five share consistent directions of regulation (over-expressed in KD: *IDH2*; under-expressed in KD: *SORL1, ITGAX, SMAP2, CD74*).

### Comparison of transcriptomic profile between human coronary artery and mice aorta homolog genes

**Figure 2:**
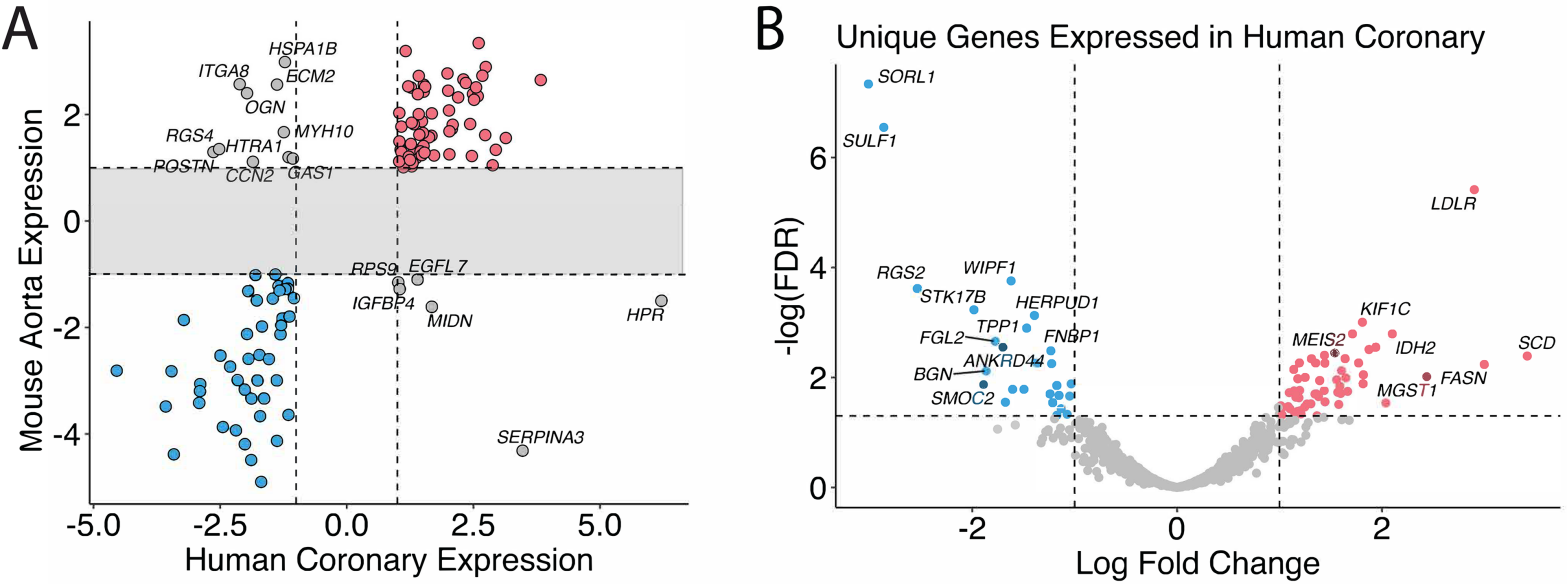
Homolog comparison between mice and humans. **(A)** Significant genes in mice and humans are matched based on their homologs. Genes that are over-expressed in KD in both humans and mice are colored in red, under-expressed in blue, and otherwise in gray. **(B)** Genes that are uniquely differentially expressed in the human coronary samples but not in the mice are shown and annotated with associated HGNC gene symbols. The genes whose expression profiles fall into the shaded area of the scatter plot are omitted in Figure 3A and shown in Figure 3B instead.

Eighty-one genes overlapped in the human coronary artery and mice aorta data, and 67 of the 81 genes were similarly up and down-regulated in both humans and mice: 30 genes were up-regulated, and 37 were down-regulated (Figure 2A, Supplementary Table 1). These include previously identified KD-significantly upregulated genes: *CD74*, *SFRP4*, *ITGA4*, and *IKZF1*. Figure 2B shows the genes in the gray zone of Figure 2A, and those are genes differentially expressed in coronary artery samples in human KD patients but not in aorta samples in the KD mouse model. There are many genes uniquely altered in human KD patients but not in the KD mouse model. The genes uniquely differentially expressed in the human KD patients are presented in Supplementary Table 2, and those in the KD mouse model are in Supplementary Table 3. In other words, gene expression differences unique to KD patients were not identified in KD mouse models, providing the potential to develop new genetic KD mouse models.

### KD alters expression levels of metabolic and cardiomyopathy-related genes

**Figure 3:**
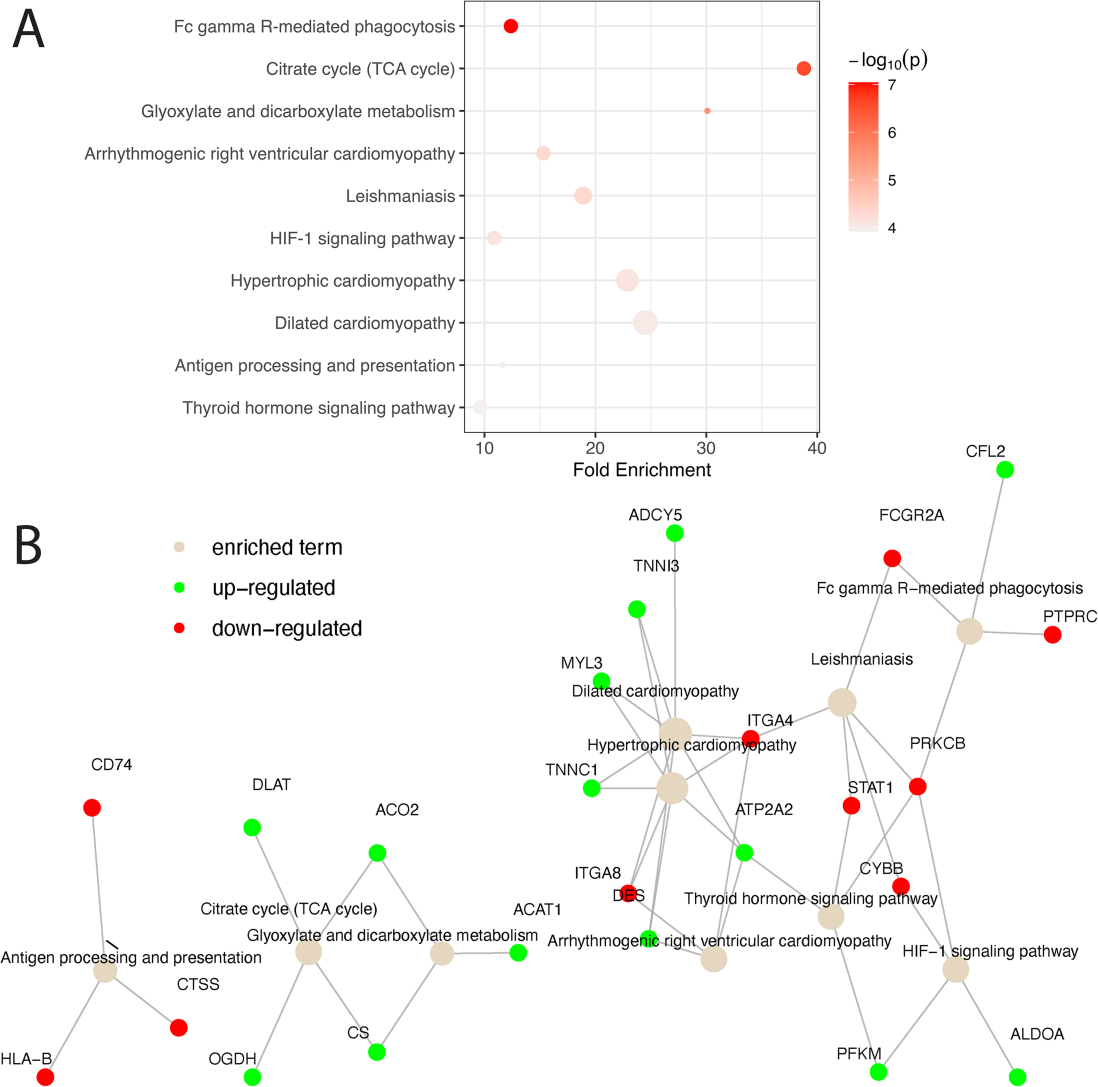
Pathway analysis of matched homologs between humans and mice. **(A)** Matched homologs that are consistently over- or under-expressed in humans and mice are examined for biological pathway enrichment. The top 10 pathways enriched are shown and ranked based on statistical significance. The color of the dots representing the enriched pathways is scaled based on their respective enrichment *P*-value and the size of the dots is scaled based on the number of genes involved in each pathway. **(B)** A network diagram is presented to showcase all significant genes that are up- (green) and down- (red) regulated in the enriched pathways (light brown). Solid gray lines connecting genes and pathway terms indicate the involvement of genes in the pathways of interest.

Biological pathway analysis of the overlap gene list revealed the involvement of *CS*, *ACO2*, *OGDH*, and *DLAT* in metabolic pathway regulation (Figure 3). In addition, genes associated with the cardiomyopathy pathway, e.g., *DES, TNNC1, TNNI3, MYL3, ADCY5, ATP2A2, ITGA4,* and *ITGA8,* were significantly differentiated between KD and non-KD counterparts. Biological pathway analysis of the genes unique to the human or the mouse model also reveals some enrichment signals of biological pathways as shown in Supplementary Table 4 (human) and Supplementary Table 5 (mouse model), suggesting potentially distinctive disease phenotypes in humans and mice.

### Transcriptomic profiles of KD in single-cell RNA sequencing studies

**Figure 4:**
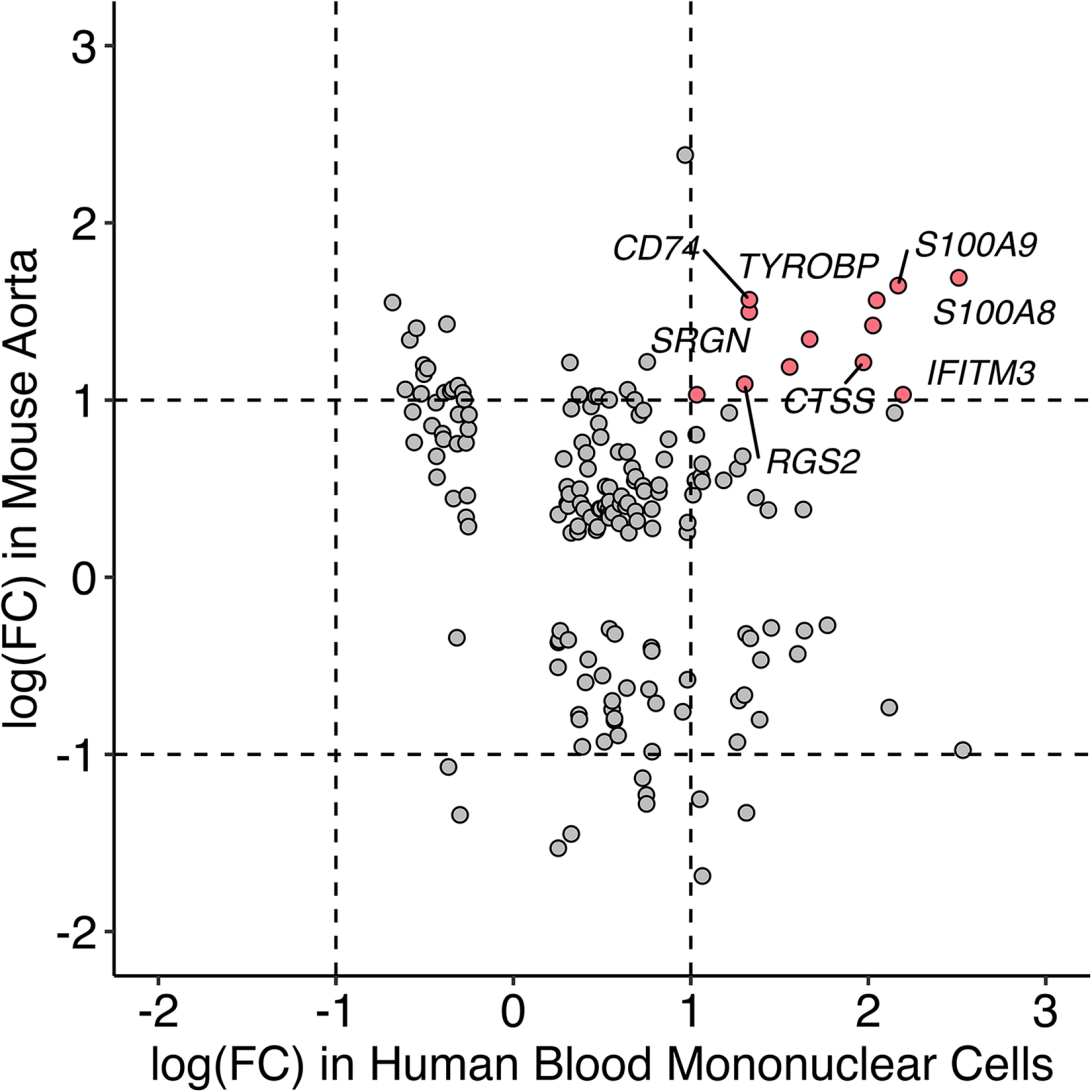
Comparison of gene expression profile between mice and humans in single-cell RNA sequencing studies. Homolog genes from single-cell RNAseq studies in mice and humans are presented based on their respective log fold change of expression. Genes in red are consistently overexpressed in both mice and humans as shown in single-cell RNAseq studies whereas the directionalities of expression of genes in gray are inconclusive.

Single-cell RNA sequencing data with human mononuclear cells and mouse aorta samples that passed quality control analyses were homogenized for differential gene expression. To increase the power of the analysis, we used entire cell populations in samples for the study. Differentially expressed genes found in the mouse aorta were converted to their human homolog and compared with findings in the human blood mononuclear cells. As shown in Figure 4, only a limited number of genes share similar directions of regulation in the mouse and human studies while reaching significant log-fold changes. Despite limited significant overlap, previously identified KD diagnostic markers, including *S100A8* and *S100A9,* are overexpressed in mice models of KD and humans with KD (49). Immune-related and interferon signaling genes, including *CD14*, *CD74*, *IFITM2,* and *IFITM3,* are also consistently overexpressed in mice models of KD and humans with KD (Table 2). Nonetheless, due to the limited read depth of the single-cell RNA sequencing datasets, conducting a comprehensive biological pathway analysis using overlapping genes between humans and mice proved to be a challenge.

**Table 2:** Consistently differentially expressed genes in humans and mice in scRNAseq studies.

Gene Name	Human		Mouse		Description
	Log(FC)	FDR	Log(FC)	FDR	
*IFITM2*	1.034	4.90E-185	1.03	1.66E-235	Interferon Induced Transmembrane Protein 2
*RGS2*	1.304	1.00E-300	1.09	1.00E-300	Regulator Of G Protein Signaling 2
*SRGN*	1.329	5.01E-196	1.497	1.77E-103	Serglycin
*CD74*	1.33	6.04E-256	1.566	1.13E-17	Cd74 Molecule
*MS4A6A*	1.557	4.73E-14	1.187	1.00E-300	Membrane Spanning 4-Domains A6A
*CD14*	1.67	1.98E-174	1.344	1.92E-236	Cd14 Molecule
*CTSS*	1.973	1.00E-300	1.213	2.24E-22	Cathepsin S
*FCER1G*	2.026	5.62E-250	1.421	1.40E-31	Fc Epsilon Receptor Ig
*TYROBP*	2.047	1.31E-159	1.563	1.72E-38	Transmembrane Immune Signaling Adaptor Tyrobp
*S100A9*	2.169	2.12E-49	1.646	1.45E-86	S100 Calcium Binding Protein A9
*IFITM3*	2.196	1.00E-300	1.03	1.66E-235	Interferon Induced Transmembrane Protein 3
*S100A8*	2.51	5.02E-147	1.69	4.63E-110	S100 Calcium Binding Protein A8

Genes that are consistently differentially expressed in humans and mice are tabulated along with their log fold changes [Log(FC)] and *P*-values after adjusted for false discovery rates (FDR).

## Discussion

Kawasaki Disease (KD) is a complex inflammatory condition that affects various tissues and organs, and understanding the gene expression patterns associated with the disease is crucial for advancing our knowledge and developing effective treatments. In this meta-analysis, we have identified several key findings regarding gene expression in KD across different tissues and species.

The tissue-specificity of gene expression in KD patients is a notable observation (Figure 1). The gene markers identified in human patients’ aorta and whole blood samples exhibit distinct profiles. Even for the same genes, the direction of regulation can differ. The current treatment for the acute phase of Kawasaki disease is aimed at preventing coronary vessel inflammation followed by coronary dilation and aneurysm because those events increase mortality in KD patients. It is thus urgent to understand the mechanisms of coronary inflammation to properly prevent coronary malfunction in KD patients. However, it is challenging to get coronary vessel samples from the patients. Collection of blood samples from those patients is relatively easier; however, our data suggest that the significant genes found in blood might not be appropriate to use for developing the treatment for coronary inflammation in KD patients due to the dissimilarity of gene profiles between human blood and coronary arteries. Therefore, relevant animal models for the KD study are required.

Among six genes that are differentially expressed and consistently regulated by KD in blood and coronary artery samples, we found over-expression of *IDH2* and under-expression of *SORL1, ITGAX, SMAP2*, and *CD74* (Figure 1C). *IDH2*, or *Isocitrate Dehydrogenase 2*, is an enzyme involved in cellular metabolism, specifically in the citric acid cycle, where it participates in energy production and maintenance of cellular redox balance (50–52). Dysregulation of the citric acid cycle and cellular metabolism can be associated with various pathological conditions, including inflammatory diseases like KD. Over-expression of *IDH2* in KD suggests a potential link between altered metabolic processes and the disease, warranting further investigation into how this contributes to KD pathogenesis. *SORL1* (53, 54)*, ITGAX* (55)*, SMAP2* (56), and *CD74* (57) are all genes that are heavily involved in the immune responses and vascular inflammation biological pathways. The downregulation of these genes may be connected to the inflammatory response and immune dysregulation seen in KD patients.

Cross-species comparisons showed a predominantly consistent direction of gene regulation between human coronary and mouse aorta cells (Figure 2, Supplementary Table 1). This similarity can be attributed to both similar tissue types used among humans and mice as well as the shared biology. Pathway enrichment analysis revealed the enrichment of the cardiomyopathy pathway, indicating the potential involvement of cardiac-related processes in KD pathogenesis. Additionally, pathways related to metabolic shifts, such as the citrate cycle (TCA cycle) and glyoxylate and dicarboxylate metabolism, were consistently affected in both humans and mice (Figure 3). These data suggests that the vascular contractility and metabolism in the vessels might be altered in KD patients. The genes involved in these cascades can thus be a great therapeutic target to minimize coronary malfunction in KD patients. The differences in gene regulation direction between species may be attributed to intrinsic biological disparities. Identifying unique human genes underscores the need to understand the underlying mechanistic differences of KD in human and animal models (Figure 2B, Supplementary Tables 2–S5). Investigating the pathophysiological role of unique human genes in KD development in mice might give us a great opportunity to reveal novel mechanisms previously missed due to the lack of information from gene profiling data in KD mouse models.

While the protective effect of female sex in the development of KD has been observed in humans, the gene expression patterns of individuals with KD do not appear to differ significantly between sexes (Supplementary Figure 1). This suggests that the protective effect might not be directly related to differential gene expression but rather to other factors that modulate disease susceptibility in a sex-specific manner. Of note is that KD patients are usually young; therefore, the hormonal factor might not play a critical role in developing KD.

Comparison of single-cell RNA sequencing studies across different tissues and species has yielded limited results due to several factors, including modest sequencing depth, small sample sizes, and the influence of tissue-specific gene expression patterns. Nevertheless, despite variations in tissue types, some genes that were previously implicated in KD, such as *S100A8, S100A9* (58–60), *CD14, CD74, IFITM2* (61, 62), *IFITM3* (63), and *ITGAX* (64) consistently exhibited overexpression in both humans with KD and the mouse model of KD. *S100A8* and *S100A9*, which encode calcium and zinc-binding proteins of the same name, are upregulated in response to immune and inflammatory challenges. Notably, research into IVIG treatment for KD has revealed elevated levels of *S100A8* and *S100A9* in the serum of KD patients during the acute phase, with a subsequent decline in levels post-treatment, except in cases where patients developed coronary aneurysms. As a result, S100A8 and S100A9 are considered promising biomarkers for diagnosing and monitoring the progression of KD in patients. Given these findings, it is imperative to delve further into the biological significance of these genes within the context of KD to gain a more comprehensive understanding of their specific roles in the disease process.

Animal models play a vital role in studying human diseases, and many studies have utilized mouse models to investigate the pathogenesis, disease progression, and potential therapeutic targets of KD and evaluate the efficacy of different treatments (41, 65). There are three most prominent ways of inducing mice with KD: intraperitoneal injections of either *Candida albicans* cell wall extract (CAWS), *Lactobacillus casei* cell wall extract (LCWE), or Nod1 ligand (FK565) (66). All three methods mimic the pathology of KD by causing aortic root inflammation and coronary arteritis. However, the CAWS model resembles KD patients most notably due to its multiinflammatory phenotypes (60). Nod1 is the least understood and researched of the three methods (66). The lack of knowledge of its mechanisms has prevented scientists from using it thus far to develop treatment methods for KD (66). Despite LCWE being more strain-independent and faster to induce in mice, its lack of KD symptom manifestations beyond the coronary arteries makes it less effective than CAWS when it comes to fully understanding the scope of KD (66). Therefore, the transcriptomic and proteomic data from other KD mouse models (e.g., CAWS) are required to compare the accuracy and reliability of animal models for KD and to advance our understanding of the disease.

The commonly significant genes identified from the human and mouse samples and with both bulk and single-cell RNA sequencing have mostly been investigated using KD animal models; however, they are still far from clinical studies. More reproducible data from different laboratories are required to move toward clinical trials. Compared to other cardiovascular diseases, there are few ongoing clinical trials for KD patients. One is defibrotide with IVIG, and the other is atorvastatin and anakinra with IVGI. Defibrotide is a mixture of single-stranded oligonucleotides, which help protect endothelial lining and reduce blood clots; however, detailed mechanisms are not known. Defibrotide is approved for use in the treatment of veno-occlusive disease in the liver. The purpose of defibrotide use with IVIG might be to smooth the blood, like a combination therapy of aspirin with IVIG for KD patients. Atorvastatin helps reduce cholesterol, and anakinra is the IL1 inhibitor, thus inhibiting inflammation. It has been known that the effect of atorvastatin is increased by anakinra. Cholesterol levels in patients with KD are not significantly changed compared to those in control patients; however, statin does show some beneficial effects in KD patients via improving endothelial and cardiac function (67). Those clinical trials are currently in Phase II, and results are expected by 2025. Further investigations on KD to find novel treatments are required.

The limitations of this study are that 1) we could not conduct further analysis using the subgroup classification (i.e., sex, age, disease severity) due to the lack of sufficient information in the original data, and 2) the comparison of the results between bulk-RNA sequencing and single-cell RNA sequencing data could not be made due to the low read depth and coverage in the single-cell RNA sequencing. These analyses require more data from public resources. In addition, the critical information missing from current knowledge is cell-type specific gene profiling from coronary vessels in KD patients or KD mouse models. Endothelial cells, smooth muscle cells, and fibroblasts play different roles in regulating vascular tone, integrity, and network formation. Understanding cell-type specific gene alterations could lead to improvements in treatment targets.

In conclusion, our meta-analysis of KD gene expression profiles highlights the tissue-specificity of gene markers in humans, the conserved gene regulation patterns between human coronary arteries and mouse aorta cells, and the enrichment of relevant pathways in KD pathogenesis. Although single-cell RNA sequencing studies yielded limited results, identifying consistent gene markers in different tissues and species can aid in developing preventative measures and treatments for KD. This underscores the critical role of animal models in understanding the complex biology and pathology of KD and emphasizes the potential for translational research to benefit children affected by this disease.

## Data Availability

Publicly available datasets were analyzed in this study. This data can be found in NCBI's GEO (https://www.ncbi.nlm.nih.gov/geo/query/acc.cgi): GSE178491, GSE64486, GSE200743, GSE152450, GSE168732, GSE141072, GSE178799, GSE178765.

## Supplementary material

Supplementary Figure S1 Bulk differential gene expression profile in mice. Volcano plot showing negative log corrected *P*-values over log fold changes of gene expression in the abdominal aorta in a mouse bulk RNAseq study in females **(A)** and males **(B)**. Genes that are over-expressed in the Kawasaki mice are colored in red and under-expressed in blue. As shown in **(C)**, most significantly differentially expressed genes (orange) distribute in the first and third quadrant. This shows that the female gene expression profile in mice is similar to the profile in the males
