# Supplementary figures and images for: Transcriptome meta-analysis of Kawasaki disease in humans and mice
Source: Front Pediatr. 2024 Sep 16;12:1423958. doi: 10.3389/fped.2024.1423958 (PMC11440715; doi:10.3389/fped.2024.1423958)

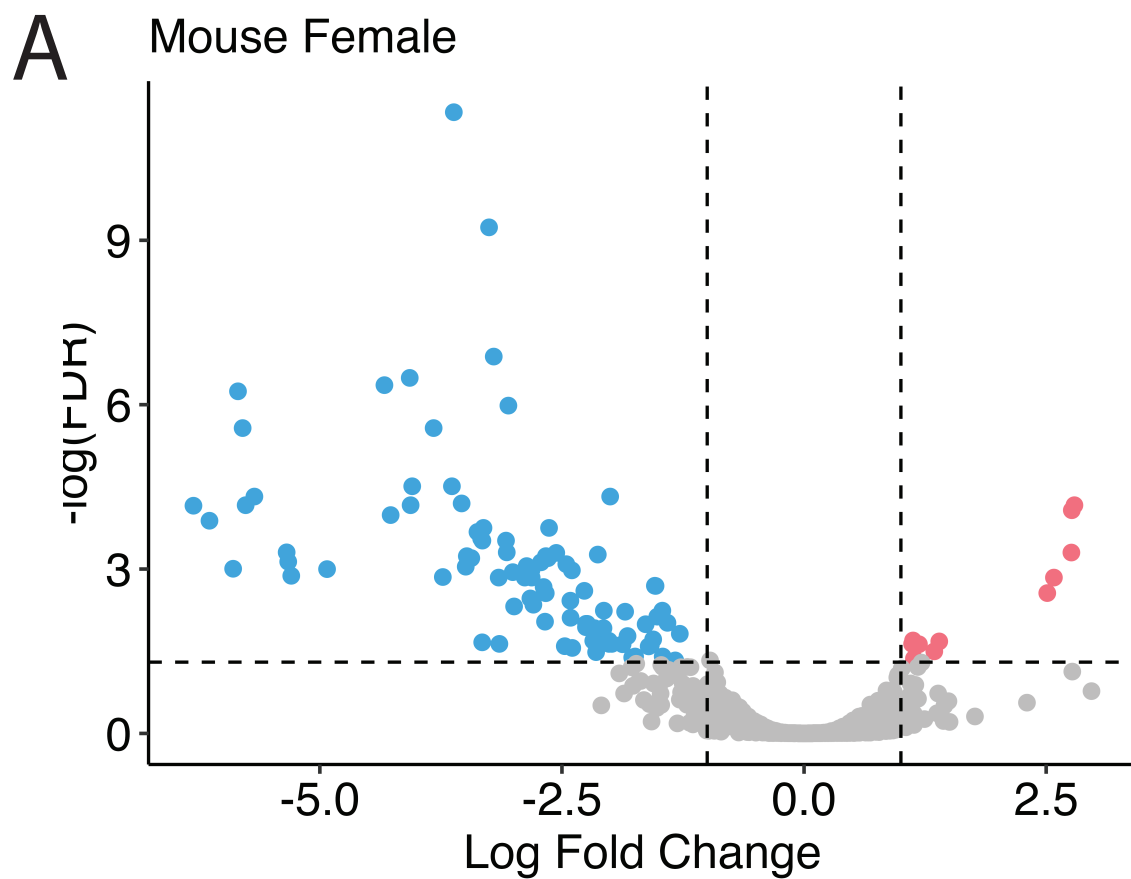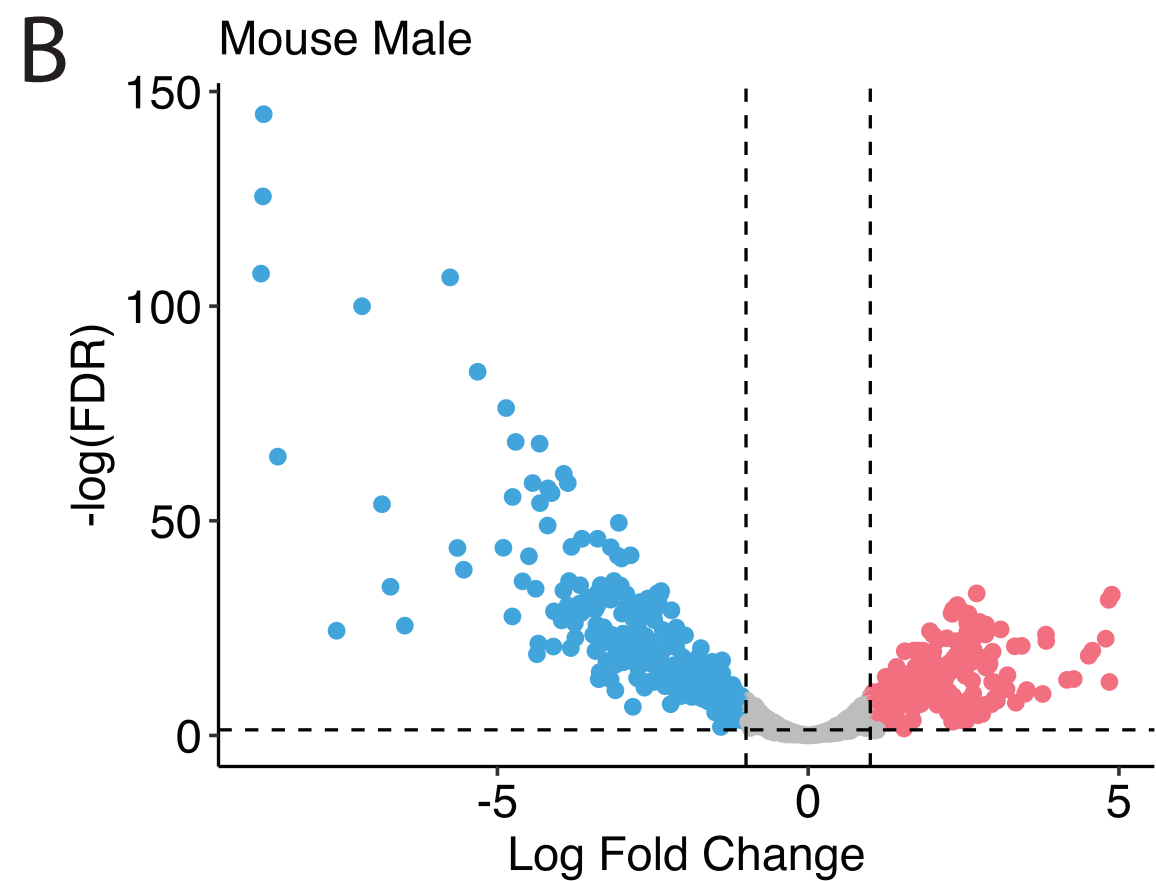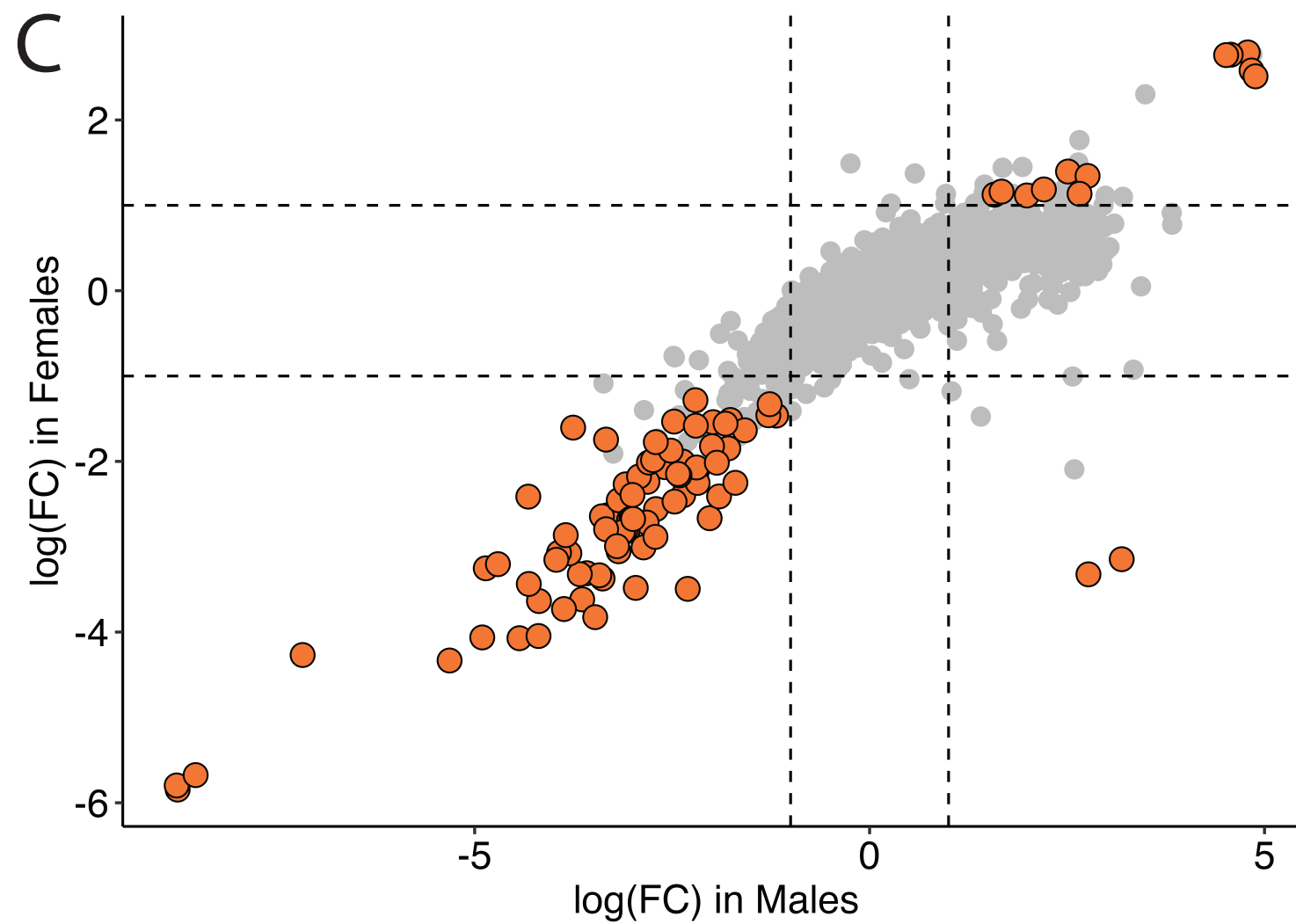

Supplement: Supplementary file 6 [file Image1.pdf]
